# Failure affects subjective estimates of cognitive load through a negative carry-over effect in virtual reality simulation of hip fracture surgery

**DOI:** 10.1186/s41077-019-0114-9

**Published:** 2019-11-21

**Authors:** Jan Duedal Rölfing, Jeppe Kempf Nørskov, Charlotte Paltved, Lars Konge, Steven Arild Wuyts Andersen

**Affiliations:** 1 Corporate HR, MidtSim, Palle Juul-Jensens Boulevard 82, 8200 Aarhus, Central Denmark Region Denmark; 2 Department of Orthopedics, Aarhus University Hospital, Aarhus, Denmark; 3 Department of Clinical Medicine, Aarhus University, Aarhus, Denmark; 4 Center for HR, Copenhagen Academy for Medical Education and Simulation (CAMES), Copenhagen, Denmark; 5 Department of Otorhinolaryngology - Head & Neck Surgery, Rigshospitalet, Copenhagen, Denmark; 6 Department of Otolaryngology, Nationwide Children’s Hospital, The Ohio State University, Columbus, OH USA

**Keywords:** Cognitive load, Hip fracture surgery, Virtual reality simulation, Surgical skills training, NASA TLX, PAAS, Orthopedic education

## Abstract

**Background:**

Cognitive overload can impair learning, and different factors might affect cognitive load during simulation-based training. In this study, we investigate the role of failure in repeated practice of virtual reality (VR) simulation of hip fracture surgery on cognitive load (CL) estimated by secondary-task reaction time test and two questionnaires.

**Methods:**

The VR simulation training program consisted of three competency levels of increasing complexity starting with the placement of a Kirschner wire in a fractured hip of one patient, adding clinical variability at the intermediate level, and performing the entire dynamic hip screw procedure in 24 different patients at the highest level. Thirteen consecutive passed simulations were required to advance to the next level. Performance was measured as passing/failing a procedure and the number of failed procedures within the latest three and five simulations. CL was measured objectively using reaction time testing during simulation and subjectively using the NASA-TLX and PAAS questionnaires. The study was carried out at a simulation center from November 2016 to March 2019. Forty-two first-year orthopedic surgery residents from the Central Denmark Region and the North Denmark Region participated in the training program.

**Results:**

A failing performance in the simulated procedure was associated with a higher CL than passing a procedure. The subjective CL estimates were affected by the number of failures during last three and five procedures with a higher number of failures being associated with a higher CL. In contrast, relative reaction time estimates of CL were not affected by previous failures.

**Conclusions:**

Questionnaires for estimation of CL seem to be affected by participant frustration after failure—a meta-cognitive “carry-over” effect. This could be a general limitation of the subjective questionnaire approach to estimate CL. Reducing CL through instructional design and handling of participant frustration might improve the learning outcome of simulation training programs.

## Background

Hip fracture surgical procedures are very common with an incidence rate of 6 per 1000 person-years in people over 65 years of age [1]. Approximately 7000 hip fracture surgeries are performed annually in Denmark, representing a key procedure for orthopedic surgeons [1]. One out of 10 hip fracture patients dies within the first months and approximately one out of four within the first year [1]. The high mortality rate is caused by a multitude of factors including the patient’s co-morbidities, the effects of immobilization due to the fracture, and suboptimal treatment and rehabilitation [1]. Ideally, a hip fracture operation will allow the patient to mobilize and fully bear weight immediately after surgery. This is key to prevent further morbidity caused by prolonged bedrest and immobilization. Conversely, intraoperative adverse events and insufficient stabilization of the fracture significantly prolong recovery and further worsen the prognosis. High-quality surgical training is therefore paramount.

Virtual reality (VR) simulation training has been introduced to address the issue of training, and previous studies have demonstrated that VR simulation training can ensure basic proficiency of the junior surgeon before continued supervised clinical training [2, 3]. Consequently, VR simulation in hip fracture surgery is available to Danish orthopedic residents at an early stage of their training. We constantly seek to improve this VR simulation training program and to explore how the learning process can be optimized. In our current simulation setting, participants have to master the basic step of placing a Kirschner wire in a fractured hip, before adding clinical variability (different fracture patterns) and complexity (full dynamic hip screw procedure).

Cognitive load theory has become a widely accepted theoretical framework in medical education [4]. This theory posits that learning is dependent on a compartmentalized cognitive architecture, consisting of working memory for processing and incorporating new information into long-term memory through the formation of mental schemata [5]. However, working memory is finite and varies with individual cognitive capacity that is highly dependent on previous schemata. The long-term learning outcome may suffer if the cognitive load (CL) of a learning task exceeds individual working memory capacity [4]. CL theory is especially relevant in medical education because of complex learning material and demands that might surpass working memory capacity [6]. CL can according to the theory be subdivided into three components: intrinsic CL describes the inherent nature—or difficulty—of the learning task; the extraneous CL relates to the instructional procedures such as presentation of new information; finally, the germane CL represents the meta-cognitive learning process with the active construction of schemata and is key in actual learning [7, 8]. CL might therefore be affected by different factors that contribute to the intrinsic, extraneous, and germane load [4, 5].

CL can be estimated by several different approaches [9]. One common method is the dual-task paradigm, where the learner is given a secondary task in which performance is measured—for example, a reaction time test. Reaction time tests are well suited because they impose very little further CL on participants; therefore, this method has been used in simulation-based surgical skills training [9–11]. Another accepted approach is to use questionnaires administered after the learning task—such as the NASA-TLX and PAAS questionnaires—to estimate CL [12, 13]. These provide a subjective estimate of the CL experienced by the participant. In simulation-based training, conflicting reports exist regarding the correlation of the different methods for CL estimation with some authors suggesting that the more extensive NASA-TLX questionnaire and the single-item PAAS questionnaire can be used interchangeably when assessing intrinsic CL, whereas others report a poor correlation [14]. Consequently, there is need for more research on the different subjective and objective estimates of cognitive load in simulation-based training and what factors might affect them.

The primary aim of this study is to investigate the relationship between failure in repeated VR simulation practice in a hip fracture surgical training program and CL estimated by secondary-task reaction time test and the NASA-TLX and PAAS questionnaires.

## Methods

### Study design and data collection

The study was a prospective educational study of a VR simulation-based training program of hip fracture surgery estimating CL with two subjective questionnaires and an objective relative reaction time test. The study flow chart is presented in Fig. 2. Participants were first introduced to the simulator by observing an instructor perform one simulation at the lowest competency level, before commencing hands-on simulation training and CL measurement.

### Participants and setting

First-year orthopedic residents employed at departments in the Central Denmark Region and the North Denmark Region were invited to participate in this study from November 2016 to March 2019. Only novices who had performed less than 10 hip fracture-related surgeries and without prior experience with the VR simulator were enrolled. The study was performed at a centralized simulation center (Corporate HR, MidtSim, Central Denmark Region, Denmark). Participants were not compensated financially for their participation.

### Simulator and metrics

**Fig. 1:**
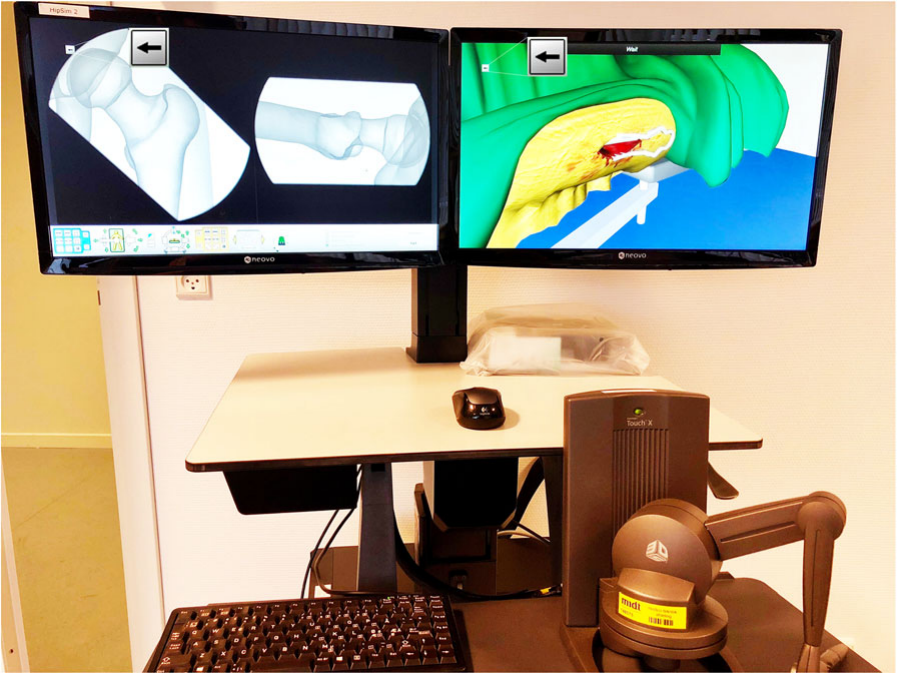
The VR hip fracture surgical simulator setup. Radiographs are acquired using two foot pedals (out of view) and displayed on the left screen. The surgical view is displayed on the right screen. Instruments are handled using a haptic device (seen in the lower right corner). Arrows indicate the secondary-task reaction time test

We used a VR surgical simulator that can simulate hip fracture surgical procedures (TraumaVision, Swemac, Sweden). Radiographs are obtained using two foot pedals (the left display: AP view to the left and lateral view to the right), and the operating field is displayed on another screen (Fig. 1). The surgical instruments are controlled through a haptic device with force feedback (Geomagic Touch X, 3D Systems, Rock Hill, SC, USA) allowing the trainee to feel the contours of the femoral shaft and the varying resistance of cortical and trabecular bone.

The simulation training program consisted of three competency levels of increasing complexity:

- Competency level 0 included only the basic simulation of a placement of a Kirschner wire in a patient model (left-sided, simple hip fracture).
- Competency level 1 introduced the clinical variability such as surgery on the left or the right side of the patient, and different fracture patterns (24 different cases).
- Competency level 2 consisted of simulation of the complete dynamic hip screw (DHS) surgical procedure (24 different cases).

In order to progress from one competency level to the next, participants had to achieve 13 successive passed procedures at every level (a “learning curve-cumulative sum” approach) [15]. For each failed test, participants were penalized by a subtraction of 7 from the amount of consecutive passed simulations. The pass/fail criteria were defined based on clinical studies and practical considerations: The main criterium, achieving a tip apex distance < 20 mm, e.g., the distance from the tip of the DHS (or Kirschner wire) to the center of the joint, has been clinically validated [16–18]. Furthermore, breach of cortical bone/cartilage into the hip joint and more than three attempts to place the Kirschner wire within the same procedure resulted in failing the test. Further details regarding the pass/fail criteria are provided as a supplement (Additional file 1).

### Outcomes

#### Performance

Performance was measured as passing or failing the simulated procedure according to the pre-defined pass/fail criteria (Additional file 1). CL outcomes were investigated in relation to the number of failures within the last three and five test attempts in the simulator.

#### Reaction time test for CL estimation

**Fig. 2:**
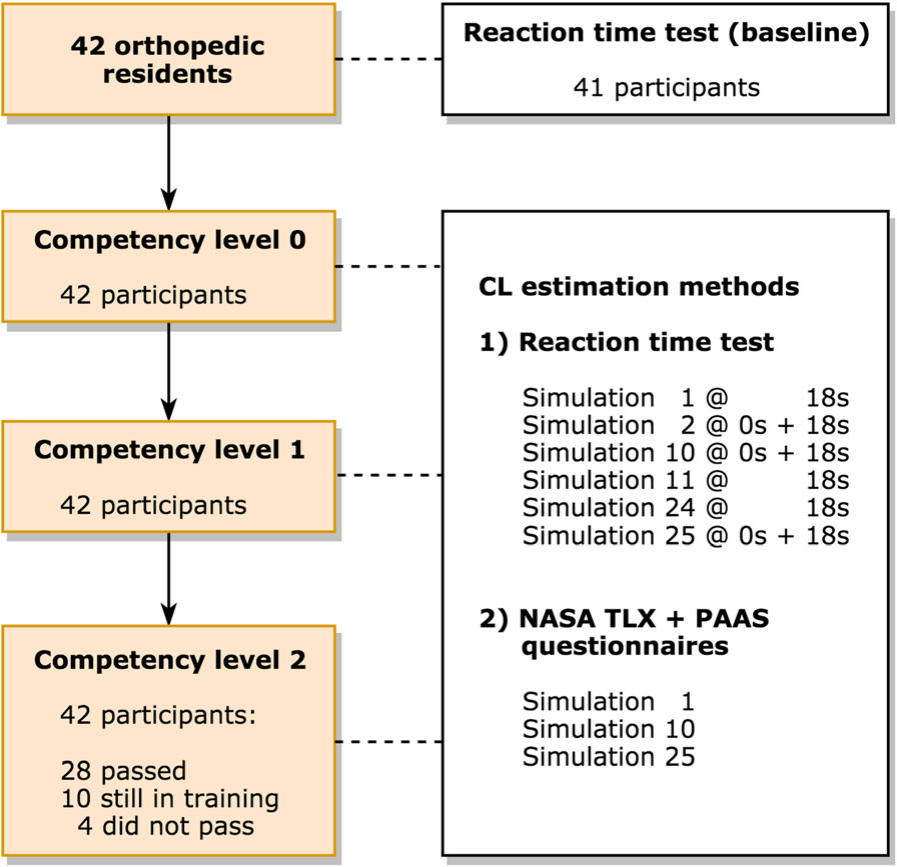
Flow chart visualizing the simulation training program on the left and outcome measurements at each competency level on the right

We used a secondary task (reaction time test) to estimate CL at predefined simulation tests at each competency level (Fig. 2). The reaction test entailed pressing an arrow (left or right) on a keyboard as fast as possible as a response to a visual cue during the simulation test at one or two different times during the test procedure (marked with arrows in Fig. 1). We similarly measured the baseline reaction time prior to each simulation training block using five measurements at varying intervals. We used the ratio of in-simulation and baseline reaction time (relative reaction time, RRT) to estimate the individual change in CL.

#### Questionnaires for CL estimation

Two validated questionnaires (NASA-TLX and PAAS) were administered after 1, 10, and 25 simulations at each competency level in order to estimate CL [12, 13]. These time points were chosen to balance not administering the questionnaires too often and still be in the training process with a likelihood of having a mix of passed and failed procedures.

The NASA-TLX questionnaire consists of six questions rated on a visual analog scale, resulting in a 0–100 point score for each question. We registered the responses in 5-point intervals. The domains represented by the six questions are mental demand, physical demand, temporal demand, performance, effort, and frustration. We chose the NASA-TLX RAW analysis method in order to estimate CL in the present study and did not weigh the different domain scores (the NASA-TLX method) [19].

The PAAS questionnaire consists of a single question in which participants ranked their mental effort in the preceding simulation procedure on a 9-point Likert scale (from very, very low mental effort to very, very high mental effort) [13].

### Sample size and statistics

The sample size was one of convenience, recruiting as many residents as possible during the study period. Data were analyzed using SPSS version 25 for MacOS X (SPSS Inc., IBM Corp., Armonk, NY). Linear mixed models were used due to a repeated measurements design. Models were built on principles for repeated measurement statistics in medical educational research as outlined by Leppink and iteratively optimized to account for relevant factors and potential interactions [20]. For all models, total procedure number was used as the repeated effect. For the reaction time data, the final models included time of measurement (0/18 s) and pass/fail in the latest simulated procedure or number of failures in the latest three or five procedures as fixed factors, respectively. For the questionnaire data, the final models included competency level (0/1/2) and pass/fail in the latest procedure or number of failures in the latest three or five procedures as fixed factors, respectively.

Due to the computerized measurement system of the reaction time, measurements where the participant did not react in time before the next reaction time measurement were assigned a reaction time of 99,999 by the system. Also, the system recorded time until the next reaction time test if the participant did not respond. Consequently, the overall distribution of the reaction time measurements was extremely right skewed. Further, a few recorded values were unrealistically low. However, censoring of extreme values would eliminate measurement of the very high cognitive load associated with missing the test. We therefore needed to define the realistic possible values of reaction time and assign the highest cutoff value for this as “penalty” for not reacting when needed. We did this similar to previous descriptions [11]. First, reaction times were log-transformed and based on the resulting frequency distribution, log(reaction time) > 4 were censored, and the remaining data was used to estimate the mean and ± 2 standard deviation (SD) of the central data. These values were next used for Winsorizing the reaction times after back transformation, resulting in reaction times < 747.1 ms being assigned the value 747.1 ms and reaction times > 4855.8 ms being assigned the value of 4855.8 ms. Finally, RRT (unitless) was calculated for each measurement.

Estimated marginal means of the linear mixed models are reported along with 95% confidence intervals (95% CI). Correlations were explored using standard linear regression. *p* values < 0.05 were considered statistically significant.

### Ethics

Ethical approval was granted by the Ethical Committee of Central Denmark Region (ref: 251/2016). This study complies with the Helsinki Declaration. All participants were informed and gave their consent that their personal data and simulator generated data were stored and sent back to them and the head of education at their department. Furthermore, consent was given to store and use anonymized data (personal data, simulator-generated data, and questionnaire data) for educational research purposes.

## Results

A total of 42 first-year orthopedic residents (26 male, 16 female; 88% right handed) were enrolled (Fig. 2). The 42 participants completed a total of 3860 simulated procedures, 244 NASA-TLX and 196 PAAS questionnaires, and 1047 reaction time tests. One of the participants did not perform the baseline reaction test; however, data of all 42 participants regarding simulated procedures and CL questionnaires were available. No participants were excluded.

The median number of procedures per participant was 90 (range 48–239), and the median total simulation time was 80 s per procedure. Of the 42 participants, 28 passed all competency levels, 10 participants have not yet completed competency level 2 at the time of study, and 4 participants did not pass competency level 2.

### CL estimated by relative reaction time

**Fig. 3:**
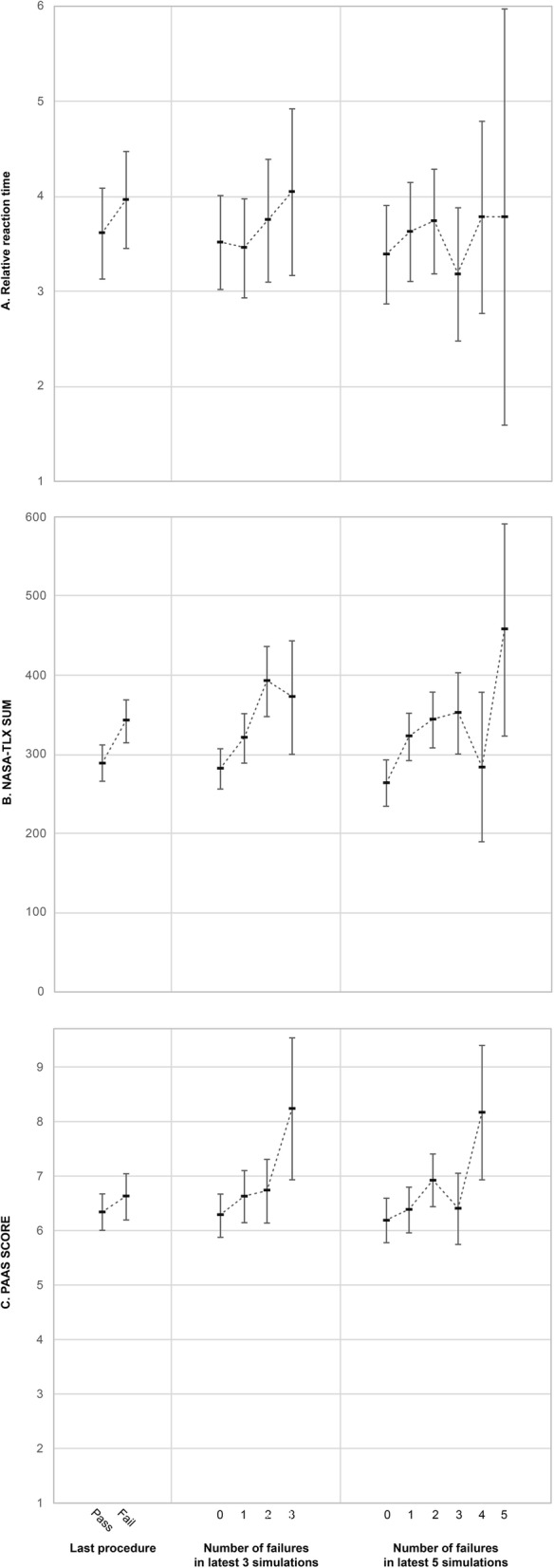
Means plot with estimated marginal means and 95% CI for **a** relative reaction time, **b** NASA-TLX score, and **c** PAAS score, according to pass/fail of the last simulation and the number of failures during the latest three test attempts and five test attempts, respectively. No PAAS questionnaires were completed with the participant having failed all five of the latest attempts

A significantly lower mean relative reaction time (RRT) was found for a passed procedure (estimated marginal mean 3.60, 95% CI [3.13–4.08]) than for failed procedures (3.96, 95% CI [3.45–4.47]) (*p* = 0.01). RRT was not affected by the number of failures within the latest three or five attempts (*p* = 0.42 and *p* = 0.35, respectively) (Fig. 3a).

### CL estimated by NASA-TLX and PAAS questionnaires

For the NASA-TLX questionnaire data, a significantly lower mean score was reported for passed procedures (estimated marginal mean 289, 95% CI [266–312]) than for failed procedures (estimated marginal mean 342, 95% CI [315–369]) (*p* < 0.0001). The number of failed procedures within the latest three and five attempts was significantly associated with higher NASA-TLX scores (*p* < 0.0001 and *p* << 0.0001, respectively) (Fig. 3b).

For the PAAS questionnaire data, a similar trend was found. The mean score for passed procedures (estimated marginal mean 6.3, 95% CI [6.0–6.7]) was lower than that for failed procedures (estimated marginal mean 6.6, 95% CI [6.2–7.0]) albeit not significantly (*p* = 0.15). This became more marked for the number of failed procedures within the latest three and five attempts (*p* = 0.006 and *p* < 0.0005, respectively) (Fig. 3c).

### Correlation between CL estimation methods

**Fig. 4:**
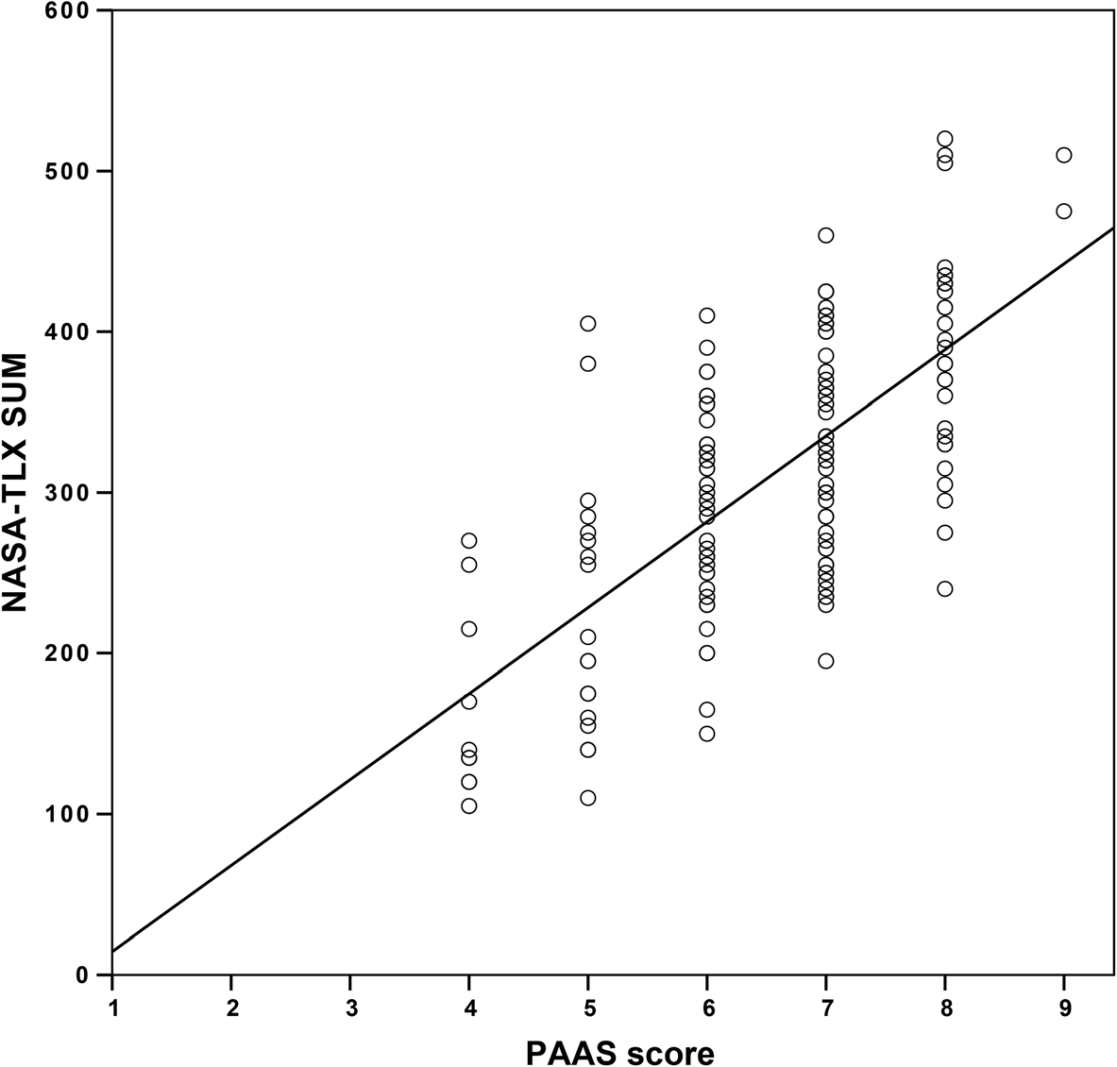
Correlation between the NASA-TLX and PAAS scores (Pearson’s r = 0.69, R^2^ = 0.47, *p* < 0.0001).

The NASA-TLX and PAAS scores significantly correlated (Pearson’s *r* = 0.69, *R*^2^ = 0.47, *p* < 0.0001) (Fig. 4). There were only very weak correlations between the RRT and the NASA-TLX and PAAS scores, respectively.

## Discussion

In this prospective educational study, we investigated cognitive load (CL) in relation to failure in VR simulation training of hip fracture surgery. We estimated CL using two different methods: an objective method consisting of secondary-task reaction time during the simulation test attempts and a subjective method consisting of two different questionnaires.

### Interpretation of results and relation with other studies

For both the objective and subjective CL estimation methods, we found that a failed simulation test was associated with a higher CL. In contrast to the reaction time measurements, the number of failed tests within the last three and five simulation test attempts were associated with higher scores on the NASA-TLX and PAAS questionnaires, reflecting higher subjective CL. This can likely be explained by the subjective method allowing a meta-cognitive carry-over effect where participants, despite being specifically instructed only to evaluate the latest procedure, seem to take previous performances into account and report a higher CL. To the best of our knowledge, this has not previously been reported in the field of medical education.

In contrast, objective methods such as secondary-task reaction time did not seem to be influenced by previous performance to the same extent. Furthermore, it also seems that RRT can measure the dynamic change of CL during a procedure because we also found participants had a 0.28 s slower reaction time at (*t* = 18 s) in the procedure when they were more cognitively engaged in the procedure than in the beginning (*t* = 0 s). This corroborates other reports on VR surgical simulation that also find that CL changes dynamically according to the complexity of the current phase of the procedure [11, 21]. Questionnaires administered after the end of the simulation can naturally not measure the change of CL during the procedure.

In contrast to another study [14], we found a substantial correlation between the NASA-TLX raw score and PAAS score. We also found a poor correlation between the CL estimated by the objective and subjective methods, which is inconsistent with a study that demonstrated some correlation between other objective measurements (pupil diameter and gaze shift rate) and NASA-TLX and PAAS scores [22]. A systematic review found that inconsistent correlations between CL and learning outcomes could relate to validity issues of CL measures and the conflicting results in the literature highlight this issue [23]. Altogether, our study adds that there might be qualitative differences in what the different estimates of CL measures.

### Strengths and limitations

One of the strengths of this study is the number of simulated procedures performed by the group of trainees with repeated measurement of CL using several well-established CL estimation methods. However, because CL was only sampled at predefined simulation attempts, only a few participants had, for example, failed four or five of the latest attempts, resulting in broad confidence intervals at these extremes.

A limitation relates to the CL estimation methods: these do not differentiate between the different cognitive load components (intrinsic, extraneous, and germane load). Consequently, we are unable to isolate which component contributes mostly to the high CL induced by VR simulation training of hip fracture surgery. This is an important consideration because not all CL is undesirable: a high germane load, for example, has positive effects on learning [24]. Nonetheless, the even higher CL found associated with failed procedures indicates that VR simulation of hip fracture surgery could benefit from considering instructional design methods that can be used to reduce CL, especially the extraneous load component that contributes little to learning [4].

### Implications

The negative “carry-over” effect as found in the questionnaire data suggests that trainees are affected by failing several procedures. This might reflect participants’ frustration since a single failed test results in a large subtraction penalty and a considerable setback in their progression in the simulation training program. This has two major implications: (1) a potential general limitation of using questionnaires for estimation of CL in medical education because a potential bias could be introduced resulting in an overestimation of actual CL. This should be considered in study design and interpretation of findings of studies using subjective methodology to estimate CL. (2) The negative carry-over effect on CL represents a negative side of providing immediate feedback and using a penalty system in competency-based simulation training. This could have particular implications in the case of repeated practice with immediate feedback by the VR simulation system. Addressing the issue of the frustration from immediate feedback and its negative effects on CL could be relevant as a future direction of study.

## Conclusions

In this study, we investigated the effects on CL of failure during a VR simulation training program of hip fracture surgery. We found that failing a simulation test was associated with a higher CL on both objective and subjective measures. Interestingly, the number of failed tests was also associated with higher subjective but not objective CL, which could be interpreted as a metacognitive “carry-over” effect: frustration of setback in progression caused participants to report a higher CL in the questionnaires. This bias should be considered when estimating CL with subjective methods such as the PAAS or RAW NASA TLX questionnaires. Finally, “frustration” management and the generally high CL induced by VR simulation training should be considered in future improvements of the training program.

## Supplementary information

**Additional file 1.** Examples of the simulator-generated feedback of passed and failed simulated procedures at different competency levels.

## Data Availability

The datasets used and/or analyzed during the current study are available from the corresponding author on reasonable request.

## Abbreviations

CL: Cognitive load
RRT: Relative reaction time
VR: Virtual reality
