# Supplementary material for: Failure affects subjective estimates of cognitive load through a negative carry-over effect in virtual reality simulation of hip fracture surgery
Source: Adv Simul (Lond). 2019 Nov 21;4:26. doi: 10.1186/s41077-019-0114-9 (PMC6868822; doi:10.1186/s41077-019-0114-9)

Example of simulator-generated feedback after a passed procedure at competency level 0

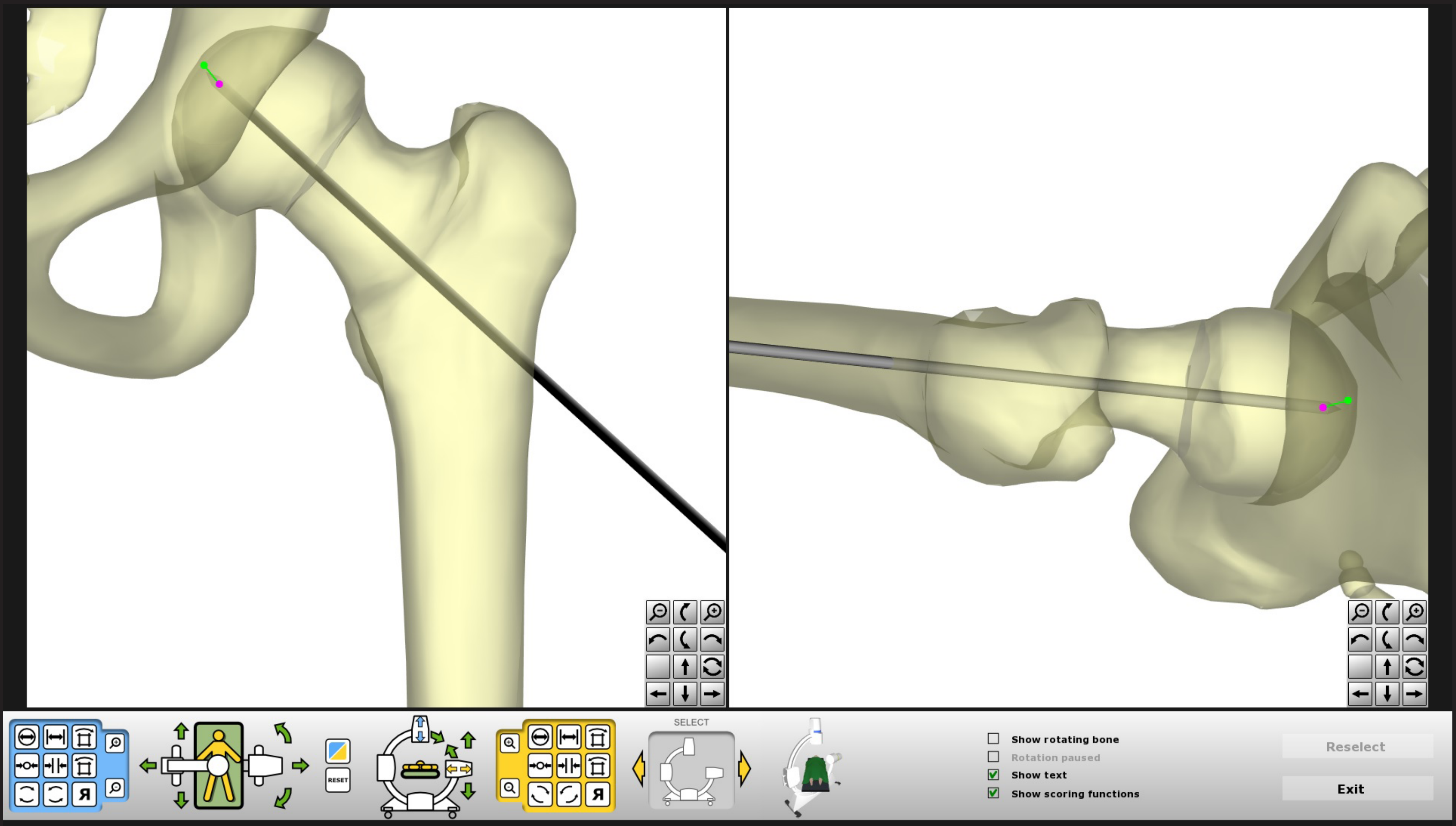

Your result: 100%   19.00 (19.00)   SUCCESS!

HipSim.dk DHS competency level 0 (Rölfing J et al.)

Use arrow keys (up/down) to step through the measured parameters

Measured parameter	Value	Acc. interval	Diff
User name: guest			
Current LC-CUSUM score (-)	1.0		
Competency level (-)	0		
No of successful procedures until next level (-)	12		
Number of procedures (today) (-)	1		
Number of procedures (at current level) (-)	76		
Current procedure			
Tip-apex distance (mm)	13.3	0.0 - 20.0	0.0
Center-center or center-inferior placement			
Tip in inferior 53% of the head in AP plane (%)	46.6	0.0 - 53.0	0.0
Tip in the mid 33% of the head in lat. plane (%)	50.1	33.0 - 66.0	0.0
No violation of the cortical bone			
Guide wire distance to femoral neck cortex (mm)	9.8	0.0 - 50.0	0.0
Lag screw distance to femoral neck cortex (mm)	6.1	0.0 - 50.0	0.0
Guide wire distance to joint surface (mm)	1.8	0.0 - 20.0	0.0
Entry point dist. to center of lateral cortex (l) (mm)	1.5	0.0 - 5.0	0.0
Guide wire angle with lateral cortex (deg)	130	126 - 134	0.0
Total time (s)	128		
Number of retries (-)	1	0 - 2	0.0
Fluoroscopy (-)	16		
Number of X-rays (-)	22		
Number of X-rays in frontal view (-)	13	1 - 1000	0.0
Number of X-rays in lateral view (-)	9	1 - 1000	0.0

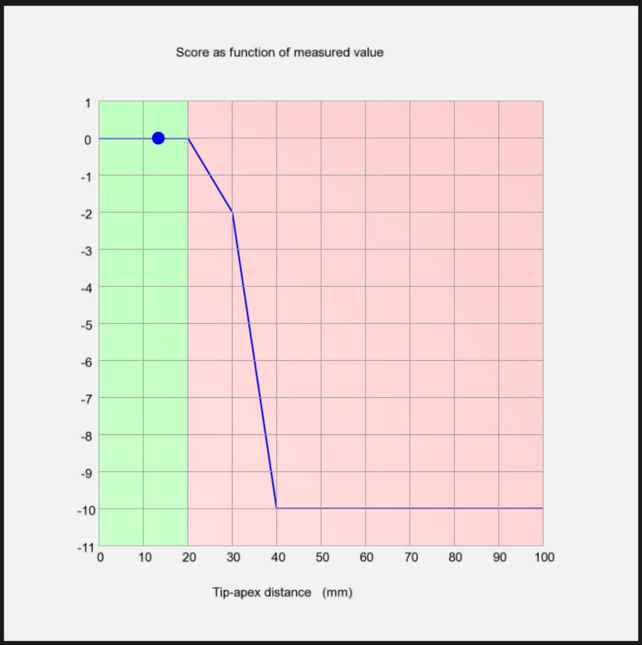

Example of simulator-generated feedback after a failed procedure at competency level 2

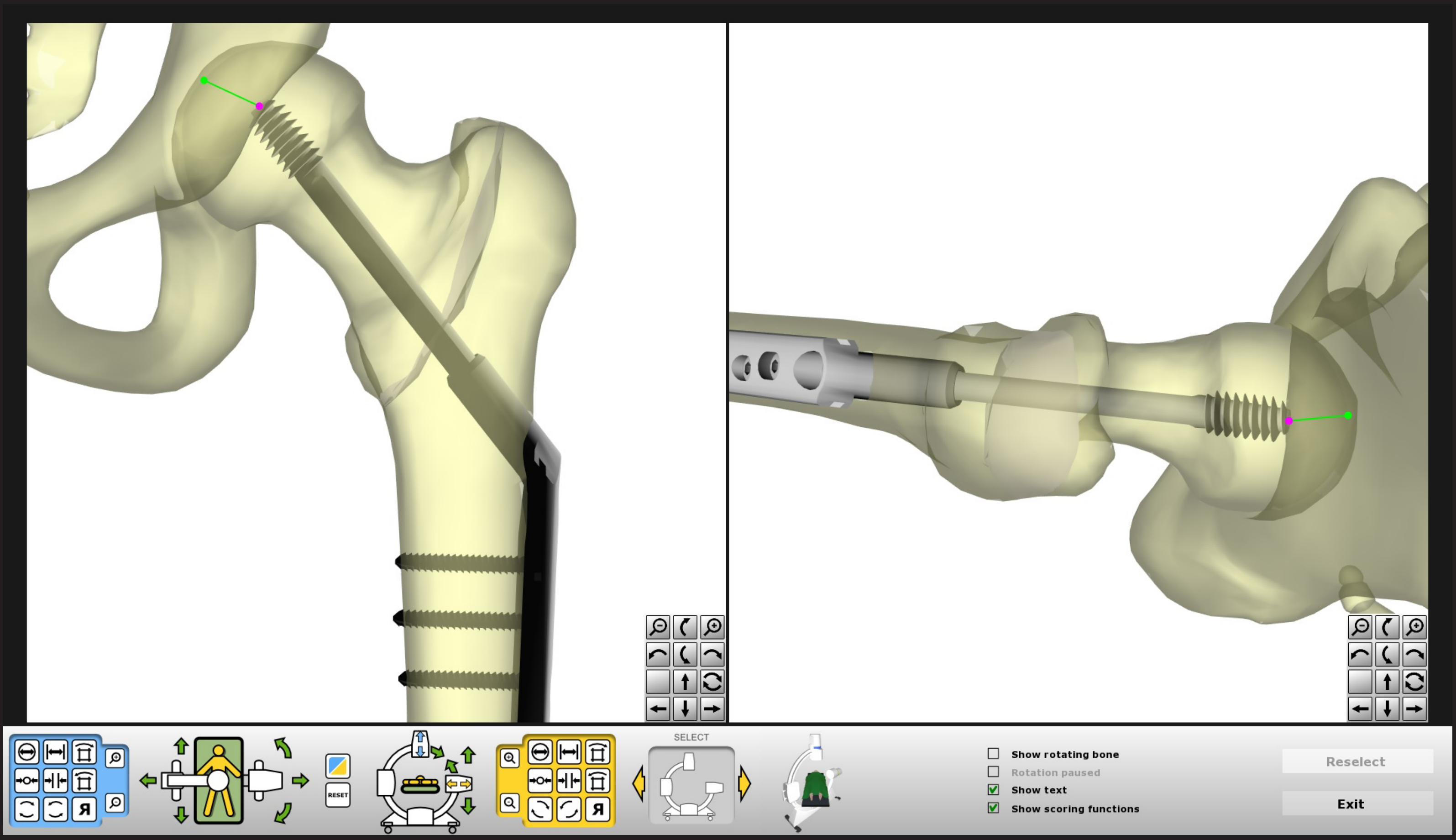

Your result: 57%   31.13 (54.00)   NO SUCCESS!

HipSim.dk DHS competency level 2 (Rölfing J et al.)

Use arrow keys (up/down) to step through the measured parameters

Measured parameter	Value	Acc. interval	Diff
User name: guest			
Current LC-CUSUM score (-)	0.0		
Competency level (-)	2		
No of successful procedures until next level (-)	13		
Number of procedures (today) (-)	5		
Number of procedures (at current level) (-)	14		
Current procedure			
Tip-apex distance (mm)	32.7	0.0 - 20.0	-4.2
Center-center or center-inferior placement			
Tip in inferior 53% of the head in AP plane (%)	57.3	0.0 - 53.0	-6.1
Tip in the mid 33% of the head in lat. plane (%)	49.9	33.0 - 66.0	0.0
No violation of the cortical bone			
Guide wire distance to femoral neck cortex (mm)	9.1	0.0 - 50.0	0.0
Lag screw distance to femoral neck cortex (mm)	5.4	0.0 - 50.0	0.0
Guide wire distance to joint surface (mm)	-27.8	0.0 - 20.0	-7.0
Step reamer distance to joint surface (mm)	12.9	2.0 - 50.0	0.0
Lag screw distance to joint surface (min.) (mm)	12.3	2.0 - 50.0	0.0
Lag screw distance to joint surface (mm)	15.4	3.0 - 10.0	-5.4
Entry point dist. to center of lateral cortex (l) (mm)	1.3	0.0 - 5.0	0.0
Plate end inside bone projection (0/1)	1.0	1.0 - 2.0	0.0
3.2 mm drill outside cortex (mm)	5.7	0.0 - 15.0	0.0
Cortical screw outside cortex (mm)	3.6	0.1 - 4.0	0.0
Lag screw length outside barrel (mm)	2.3	-9.9 - 2.0	-0.2
Guide wire angle with lateral cortex (deg)	140	136 - 144	0.0
Plate angle (deg)	140		
Total time (s)	126		
Number of retries (-)	0	0 - 2	0.0
Fluoroscopy (-)	15		
Number of X-rays (-)	34		
Number of X-rays in frontal view (-)	21	1 - 1000	0.0
Number of X-rays in lateral view (-)	13	1 - 1000	0.0

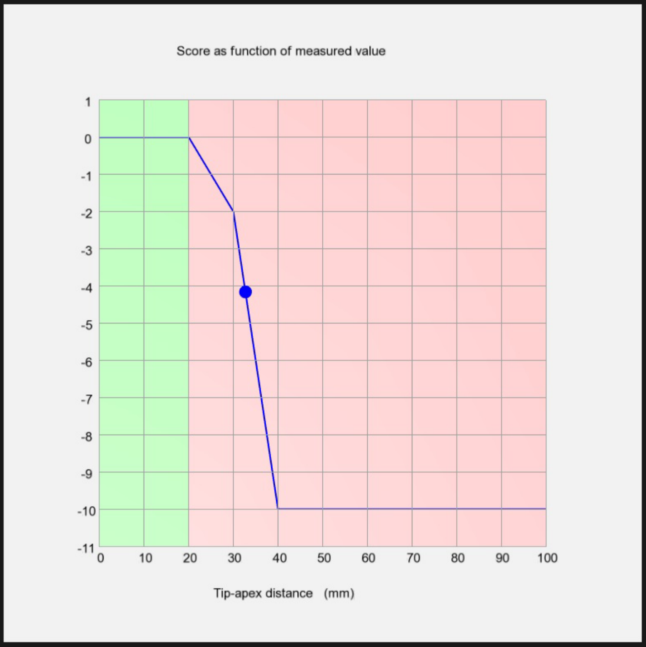

Supplement: Supplementary file 1 — Additional file 1. Examples of the simulator-generated feedback of passed and failed simulated procedures at different competency levels. [file 41077_2019_114_MOESM1_ESM.pdf]
